# Temporal and Contextual Variations in Job Satisfaction Between Physicians and Nurses: A Systematic Review and Meta-Analysis

**DOI:** 10.3390/healthcare13233008

**Published:** 2025-11-21

**Authors:** Nazerke Narymbayeva, Maksut Kamaliev, Konrad Tomasz Juszkiewicz, Kuralay Kanafyanova, Sholpan Aliyeva, Nadira Aitambayeva, Laila Nazarova, Sharapat Moiynbayeva, Akylbek Saktapov, Shnara Svetlanova

**Affiliations:** 1 Department of Healthcare Management, Kazakhstan Medical University “KSPH”, Almaty 050060, Kazakhstan; drkamaliev@gmail.com; 2 Health Department, Royal Tropical Institute, 1092 AD Amsterdam, The Netherlands; kjuszkiewicz12@yahoo.com; 3 Emergency Department and Hospitalization Bureau Portal, Kazakh Institute of Oncology and Radiology, Almaty 050022, Kazakhstan; kuralayk73@gmail.com; 4 Department of Gynecology, National Hospital of the Medical Center of the Presidential Administration, Almaty 050000, Kazakhstan; tansholpan2509@gmail.com; 5 Department of Public Health and Social Sciences, Kazakhstan Medical University “KSPH”, Almaty 050060, Kazakhstan; aitambaeva.nadira@gmail.com; 6 Department of Epidemiology, Biostatistics and EBM, Kazakhstan Medical University “KSPH”, Almaty 050060, Kazakhstan; zauirbekovna92@gmail.com; 7 Department of Science and Consulting, Kazakhstan Medical University “KSPH”, Almaty 050060, Kazakhstan; sharapat.lek@gmail.com; 8 Department of Epidemiology, Biostatistics and Evidence-Based Medicine, Al-Farabi Kazakh National University, Almaty 050040, Kazakhstan; akylbekzone@gmail.com; 9 Department of Public Health, Asfendiyarov Kazakh National Medical University, Almaty 050012, Kazakhstan; svetlanova.sh@kaznmu.kz; 10 Department of Nursing, Kazakhstan Medical University “KSPH”, Almaty 050060, Kazakhstan

**Keywords:** job satisfaction, physicians, nurses, COVID-19 pandemic, healthcare settings, emergency care, primary care, meta-analysis, systematic review, workforce well-being, interprofessional collaboration

## Abstract

**Highlights:**

**What are the main findings?**

- No consistent or clinically meaningful differences in job satisfaction were found between physicians and nurses across the COVID-19 periods.
- Setting-specific patterns emerged: physicians reported slightly higher satisfaction in emergency care during the pandemic and in mixed settings post-pandemic, while nurses reported higher satisfaction in primary care after the pandemic.

**What are the implications of the main findings?**

- Job satisfaction is shaped more by organizational context, workload, and historical timing than by professional role alone.
- Efforts to enhance workforce well-being should prioritize modifiable factors—such as workload management, staffing adequacy, and interprofessional collaboration—tailored to specific care environments.

**Abstract:**

**Objectives**: This systematic review and meta-analysis evaluated differences in job satisfaction scores between nurses and physicians, examining variation by (a) care setting (hospital, emergency department, outpatient, mixed), and (b) time period (pre-COVID, during COVID, post-COVID). **Methods**: We systematically searched PubMed, Scopus, ScienceDirect, Web of Science, and CINAHL for studies published between January 2020 and July 2025. Eligible studies reported mean and standard deviation values for job satisfaction among physicians and nurses in healthcare settings across the specified timeframes. Studies were excluded if they assessed other types of satisfaction or combined data across COVID periods. Pooled standardized mean difference (SMD) was calculated using random-effects models in R. **Results**: Before COVID-19, the SMD was −2.40 (95% CI −8.05 to 3.26; I^2^ = 98%). During the pandemic, the estimate was 1.39 (95% CI −0.57 to 3.35; I^2^ = 91.5%), and post-pandemic, it remained small (SMD = 0.29; 95% CI −1.63 to 2.22; I^2^ = 95.8%). Emergency care during COVID showed a significant advantage for physicians (SMD = 0.29; 95% CI 0.05 to 0.52; I^2^ = 0%). Post-COVID, mixed settings slightly favored physicians (SMD = 0.06), while primary care favored nurses (SMD = −0.30); subgroup differences were significant. **Conclusions**: The findings reveal that job satisfaction is not solely determined by professional role but is significantly influenced by temporal and contextual factors. Job satisfaction is shaped more by temporal and contextual factors than by professional role. While no consistent differences were observed pre-pandemic, emergency care favored physicians during COVID, and post-pandemic trends showed modest advantages for nurses in primary care and physicians in mixed settings. Due to the methodological limitations of this meta-analysis, including high heterogeneity, reliance on cross-sectional data, and very low/low certainty of evidence, these results should be interpreted with caution.

## 1. Introduction

Job satisfaction among healthcare professionals is a critical determinant of workforce stability, care quality, and patient outcomes [1]. In recent years, increasing attention has been directed toward understanding the psychosocial and organizational factors that influence job satisfaction in clinical settings, particularly among nurses and physicians who constitute the backbone of healthcare delivery [2,3]. Persistent staffing shortages, high turnover rates, and administrative overload have underscored the urgency of addressing job satisfaction as a strategic priority for health systems worldwide [4,5].

Evidence from multiple studies indicates that strong nurse–physician collaboration is positively associated with higher levels of job satisfaction for both groups [6,7,8]. Conversely, elevated organizational commitment, when not matched by adequate support, has been linked to increased burnout among healthcare providers [9]. Although nurses and physicians share similarly demanding work conditions, their roles, responsibilities, and professional expectations differ significantly, potentially resulting in distinct experiences of job satisfaction [4,5]. These disparities may be further influenced by the specific setting of care provision, such as hospitals, emergency departments, outpatient clinics, or mixed environments, each of which presents unique operational challenges and interpersonal dynamics [10,11]. Notably, evidence suggests that job satisfaction levels among emergency department staff vary between private and public hospitals, highlighting the influence of institutional context on professional well-being [12].

Additionally, the COVID-19 pandemic has introduced unprecedented stressors, fundamentally altering clinical workflows, resource distribution, and emotional labor across all levels of care [13,14,15,16]. A recent systematic review and meta-analysis on job satisfaction among healthcare providers found that high levels of work-related stress and burnout were significantly associated with lower job satisfaction [17]. These findings support the hypothesis that temporal factors—specifically the pre-COVID, during-COVID, and post-COVID periods—may play a critical role in shaping job satisfaction trajectories among healthcare professionals. Despite a growing body of literature examining job satisfaction in healthcare, existing studies often focus on single professions, isolated settings, or narrow timeframes, limiting the generalizability of findings. To date, no comprehensive meta-analytic review has systematically quantified the direct differences in job satisfaction scores between nurses and physicians across varied healthcare settings and distinct temporal phases associated with the COVID-19 pandemic.

This systematic review and meta-analysis aim to address this gap by evaluating the mean difference in job satisfaction scores between nurses and physicians. This study examines whether this difference varies according to (a) contextual factors, such as care setting (hospital, emergency department, outpatient, or mixed) and country income level, and (b) temporal factors, including the time period (pre-COVID, during COVID, and post-COVID).

## 2. Materials and Methods

### 2.1. Study Registration

This systematic review and meta-analysis of published studies was conducted according to the Preferred Reporting Items for Systematic Reviews and Meta-Analyses (PRISMA) guidelines [18]. The review protocol was submitted to PROSPERO (ID: CRD420251139056) after confirming that no similar reviews existed in the database.

### 2.2. Search Strategy

The following databases were searched for this systematic review: PubMed, Scopus, ScienceDirect, Web of Science, and CINAHL. Gray literature sources were not included in this review. The applied filters included: publication in English, inclusion in scholarly journals, and document types restricted to articles, research articles, short surveys, and short communication pieces. Where relevant, source types were limited to academic journals The search period spanned from January 2020 to July 2025. Searches were conducted on 29 July 2025, and updated on 31 July 2025, to ensure completeness. Based on a preliminary search conducted in PubMed, the following keywords were incorporated into the search strategy: (“job satisfaction” [MeSH] OR “occupational satisfaction”) AND (“nurses” [MeSH]) AND (“physicians” [MeSH] OR “medical staff” [MeSH]) AND NOT review). The complete PubMed search string is available in Supplementary Table S1.

### 2.3. Eligibility Criteria, Study Selection and Data Collection

Table 1 outlines the inclusion and exclusion criteria for the study selection process, based on the Population, Exposure, Comparator, Outcome, and Study Design (PECOS) framework.

The assessment of eligibility criteria and data collection were conducted in accordance with the Preferred Reporting Items for Systematic Reviews and Meta-Analyses (PRISMA) guidelines [18]. Two independent researchers (N.N. and N.A.) performed a standardized search. After completing the database searches, all references were imported into Mendeley for duplicate removal. Subsequently, only unique records were screened against the eligibility criteria based on titles and abstracts. In the second stage of the eligibility assessment, the full texts of selected articles were evaluated according to the inclusion criteria. A standardized data collection form was developed to extract relevant information from the included studies. Extracted data included the first author’s last name, year of publication, country, study design, assessment period (specified by year and month or categorized as pre-COVID, COVID, or post-COVID), assessment setting (hospital, emergency department, outpatient, primary care, or mixed), assessment instrument, total number of nurses and physicians evaluated, and mean and standard deviation scores on the job satisfaction scale. Two authors independently collected the data, and their respective datasheets were compared and merged. Any discrepancies in study selection were resolved through discussion with a third author (M.K.), and consensus was reached for all included studies. For studies with missing or incomplete outcome data, or without full-text availability, corresponding authors were contacted twice via email. If no response was received, the study was excluded from the final analysis. No data were imputed at any stage of this review.

### 2.4. Meta-Analysis

The meta-analysis for this study was conducted in R using RStudio, employing the meta and metafor packages (version 4.3.2) [19]. The pooled standardized mean difference (SMD) in job satisfaction scores between nurses and physicians, along with the corresponding 95% confidence interval (CI), was estimated using a random-effects model for each assessment period: pre-COVID, during COVID, and post-COVID [20]. Meta-analysis results were visually presented using forest plots, and heterogeneity was assessed by calculating I^2^ and τ^2^ statistics [21]. Potential sources of heterogeneity were explored through meta-regression based on year of publication, as well as through influence analysis and leave-one-out analysis. Publication bias was evaluated through visual inspection of funnel plots and confirmed using Egger’s test [20]. Subgroup analysis was performed based on the assessment setting, including hospital, emergency department, outpatient or primary care, and mixed settings and country income levels (World Bank income classification: high income, upper-middle income, and lower-middle income).

### 2.5. Risk of Bias

To evaluate the methodological quality and potential risk of bias in the included cross-sectional studies, an adapted version of the Newcastle–Ottawa Scale (NOS) specifically tailored for cross-sectional designs was employed. This modified tool assesses studies across three key domains: Selection, Comparability, and Outcome. The Selection domain comprises four items that examine aspects such as the representativeness of the sample, sample size justification, non-respondent handling, and ascertainment of the exposure, with a maximum score of five points. The Comparability domain includes one item that evaluates the extent to which the study controls for confounding variables, contributing up to one point. The Outcome domain consists of two items assessing the reliability of outcome measurement and the appropriateness of statistical analysis, with a maximum score of three points. The cumulative score ranges from 0 to 9, where higher scores reflect stronger methodological rigor and lower risk of bias.

Two reviewers independently conducted the quality assessments after reaching consensus on the evaluation criteria and procedures. Any disagreements were resolved through discussion. A third reviewer was involved to calculate inter-rater reliability using the intraclass correlation coefficient (ICC = 0.84), indicating strong agreement and ensuring consistency in scoring [22,23].

### 2.6. Certainty of Evidence Evaluation

In alignment with the methodological standards outlined in the Cochrane Handbook for Systematic Reviews of Interventions, the certainty of evidence for each outcome was appraised using the Grading of Recommendations Assessment, Development, and Evaluation (GRADE) framework [24]. To ensure rigor and consistency, guidance from recent methodological literature on applying GRADE in systematic reviews was also incorporated [25]. The evaluation was conducted in R using RStudio. The GRADE framework considers five domains: risk of bias, assessed with the modified Newcastle–Ottawa Scale (NOS) for cross-sectional studies across selection, comparability, and outcome criteria; inconsistency, quantified using the I^2^ statistic to measure heterogeneity; indirectness, judged through the alignment of populations, interventions, comparators, and outcomes with the PECOS framework; imprecision, determined by whether the 95% confidence interval of the pooled effect estimate crosses a predefined threshold of clinical or practical relevance; and publication bias, investigated through Egger’s regression test results.

## 3. Results

### 3.1. Study Selection and Characteristics of the Included Studies

**Figure 1:**
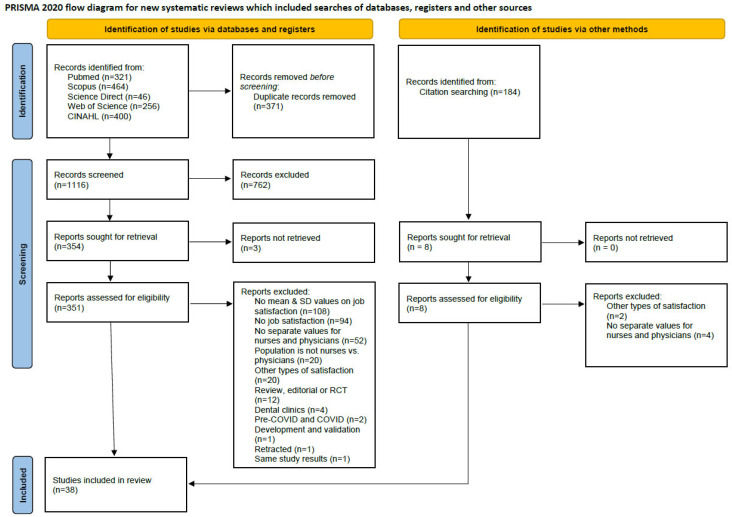
PRISMA flowchart of study inclusion.

A total of 1487 articles were identified through the systematic literature search. After removing duplicates, 1116 titles and abstracts were screened, of which 354 articles were selected for the second stage of eligibility evaluation. Full texts were unavailable for three studies and could not be assessed further. Following full-text review, 36 studies met the prespecified PECOS eligibility criteria and were included in the present meta-analysis. Among the excluded articles, 108 did not report mean and standard deviation values for job satisfaction, 94 lacked any data on job satisfaction, 52 did not present separate values required for the analysis, 20 involved populations other than physicians and nurses, 20 assessed other forms of satisfaction such as compulsion satisfaction or job dissatisfaction, 12 were reviews, editorials, or interventional studies, 4 focused on dental clinics, 2 presented combined data for pre-COVID and COVID periods, 1 was a validation study [26], 1 article had been retracted [27], and 1 study duplicated results already included in the analysis [28]. An additional 184 citations were screened through reference lists of included studies, and 2 more studies met the inclusion criteria. In total, 38 studies were included in the final meta-analysis. The PRISMA flowchart illustrating the study selection and inclusion process is presented in Figure 1 [18].

Table 2 provides a detailed summary of the studies included in this systematic review and meta-analysis, organized by assessment period: pre-COVID, during COVID, and post-COVID. All studies employed a cross-sectional design and were conducted across a wide range of geographical regions, including Asia, Europe, North America, Africa, and Oceania. The assessment periods spanned from 2020 to 2025, allowing for the examination of temporal trends in job satisfaction among healthcare professionals across different phases of the COVID-19 pandemic. Notably, the study by Chen, 2024 [29] is presented twice in the table, as it reports data for both the COVID and post-COVID periods. Several studies included subgroup descriptions to differentiate between specific populations analyzed in the meta-analysis, such as gender, specialty, departmental affiliation or assessment period. Job satisfaction was assessed using a variety of validated instruments, encompassing both globally recognized and region-specific tools. Commonly employed measures included single-item questions, the Minnesota Satisfaction Questionnaire (MSQ), the Job Satisfaction Survey (JSS), the Copenhagen Psychosocial Questionnaire (COPSOQ), the Safety Attitudes Questionnaire (SAQ), and the Warr–Cook–Wall job satisfaction scale.

### 3.2. Meta-Analysis Results

**Figure 2:**
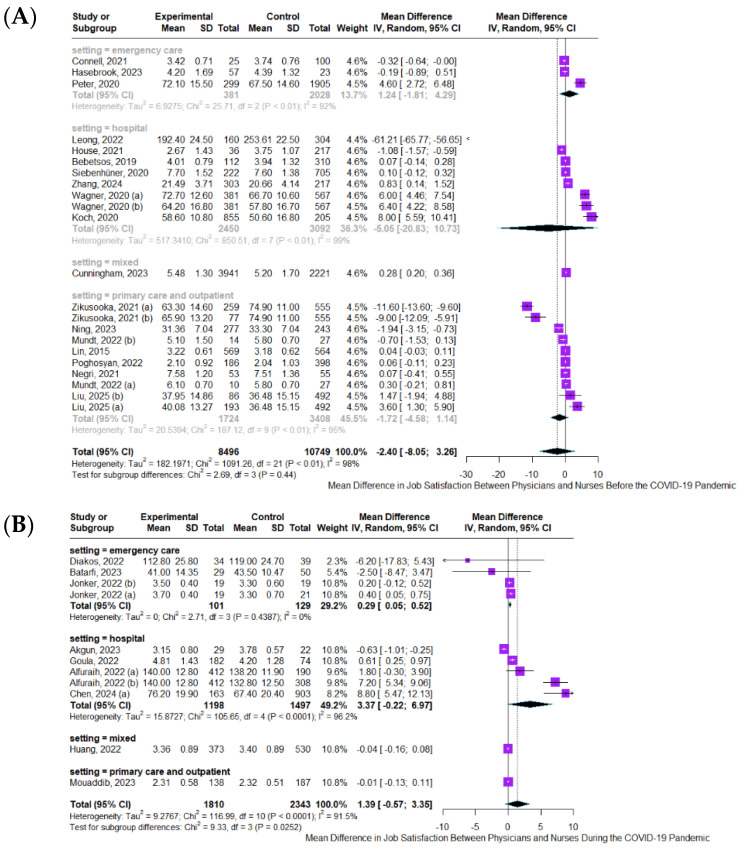

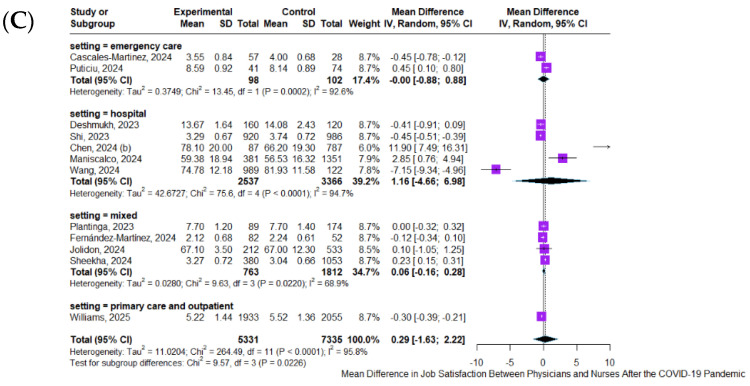
Forest plots comparing mean differences in job satisfaction between physicians and nurses across pandemic periods by job setting: (**A**) Pre-COVID-19 period; (**B**) COVID-19 period; (**C**) Post-COVID-19 period. References: Lin, 2015 [30]; Bebetsos, 2019 [31]; Koch, 2020 [32]; Peter, 2020 [33]; Siebenhüner, 2020 [34]; Wagner, 2020 [35]; Connell, 2021 [36]; House, 2021 [37]; Negri, 2021 [38]; Zikusooka, 2021 [39]; Leong, 2022 [40]; Mundt, 2022 [41]; Poghosyan, 2022 [42]; Cunningham, 2023 [43]; Hasebrook, 2023 [44]; Ning, 2023 [45]; Zhang, 2024 [46]; Liu, 2025 [47]; Alfuraih, 2022 [48]; Diakos, 2022 [49]; Goula, 2022 [50]; Huang, 2022 [51]; Jonker, 2022 [52]; Akgun, 2023 [53]; Batarfi, 2023 [54]; Mouaddib, 2023 [55]; Chen, 2024 [29]; Deshmukh, 2023 [56]; Plantinga, 2023 [57]; Shi, 2023 [58]; Cascales-Martinez, 2024 [59]; Chen, 2024 [29]; Fernández-Martínez, 2024 [60]; Jolidon, 2024 [61]; Maniscalco, 2024 [62]; Puticiu, 2024 [63]; Sheekha, 2024 [64]; Wang, 2024 [65]; Williams, 2025 [66].

The results of the meta-analysis assessing differences in job satisfaction between physicians and nurses, stratified by pandemic period and clinical setting, are presented in Figure 2. Panel A presents the results of the meta-analysis assessing differences in job satisfaction between physicians and nurses before the COVID-19 pandemic. The overall pooled SMD, calculated using a random-effects model, was −2.40 (95% CI: −8.05 to 3.26; I^2^ = 98%; *p* < 0.01), indicating substantial heterogeneity across studies. In the emergency care subgroup, the pooled SMD was −1.24 (95% CI: −4.81 to 4.29; I^2^ = 92%; *p* < 0.01), while hospital-based studies reported a slightly higher SMD of 3.35 (95% CI: −2.08 to 10.73; I^2^ = 99%; *p* < 0.01), both reflecting very high heterogeneity. The mixed-setting subgroup yielded a SMD of 0.28 (95% CI: 0.20 to 0.36; I^2^ = 0%), representing the only statistically significant result favoring physicians. Primary care and outpatient studies showed a pooled SMD of −2.72 (95% CI: −6.58 to 1.14; I^2^ = 97%; *p* < 0.01), suggesting lower job satisfaction among nurses, though without statistical significance. The test for subgroup differences was not statistically significant (χ^2^ = 2.69, df = 3; *p* = 0.44), indicating that the type of healthcare setting did not systematically influence job satisfaction differences in the pre-pandemic period. Panel B presents the pooled results during the COVID-19 pandemic. The overall pooled SMD, calculated using a random-effects model to account for between-study heterogeneity, was 1.39 (95% CI: −0.57 to 3.35; I^2^ = 91.5%; *p* < 0.01), indicating substantial heterogeneity. In the emergency care subgroup, the pooled SMD was 0.29 (95% CI: 0.05 to 0.52; I^2^ = 0%; *p* = 0.44), suggesting a statistically significant but modest difference favoring physicians with no observed heterogeneity. In hospital-based studies, the pooled SMD was higher at 3.37 (95% CI: −0.22 to 6.97; I^2^ = 96.2%; *p* < 0.01), though the result did not reach statistical significance due to wide confidence intervals. The mixed-setting subgroup reported a SMD of −0.04 (95% CI: −0.16 to 0.08), while primary care and outpatient studies showed a SMD of −0.01 (95% CI: −0.13 to 0.11), both indicating no significant difference between physicians and nurses. The test for subgroup differences was statistically significant (χ^2^ = 9.33, df = 3; *p* = 0.0252), suggesting that the type of healthcare setting influenced job satisfaction differences during the pandemic. Panel C shows the pooled results after the COVID-19 pandemic. The overall pooled SMD, calculated using a random-effects model, was 0.29 (95% CI: −1.63 to 2.22; I^2^ = 95.8%; *p* < 0.01), indicating substantial heterogeneity across studies. In the emergency care subgroup, the pooled SMD was 0.00 (95% CI: −0.88 to 0.88; I^2^ = 92.6%; *p* = 0.002), suggesting no difference in job satisfaction between physicians and nurses. In hospital-based studies, the pooled SMD was 1.16 (95% CI: −4.66 to 6.98; I^2^ = 94.7%; *p* < 0.01), reflecting very high heterogeneity and a non-significant result due to wide confidence intervals. The mixed-setting subgroup reported a small but statistically significant SMD of 0.06 (95% CI: 0.16 to 0.28; I^2^ = 68.9%; *p* = 0.02), indicating slightly higher job satisfaction among physicians. Primary care and outpatient studies showed a pooled SMD of −0.30 (95% CI: −0.39 to −0.21), favoring nurses and reaching statistical significance. The test for subgroup differences was statistically significant (χ^2^ = 9.57, df = 3; *p* = 0.0226), indicating that the type of healthcare setting may have influenced job satisfaction differences in the post-pandemic period.

The results of the meta-analysis assessing differences in job satisfaction between physicians and nurses, stratified by country income level, are presented in Supplementary Figure S1.

### 3.3. Meta-Regression by Publication Year

**Figure 3:**
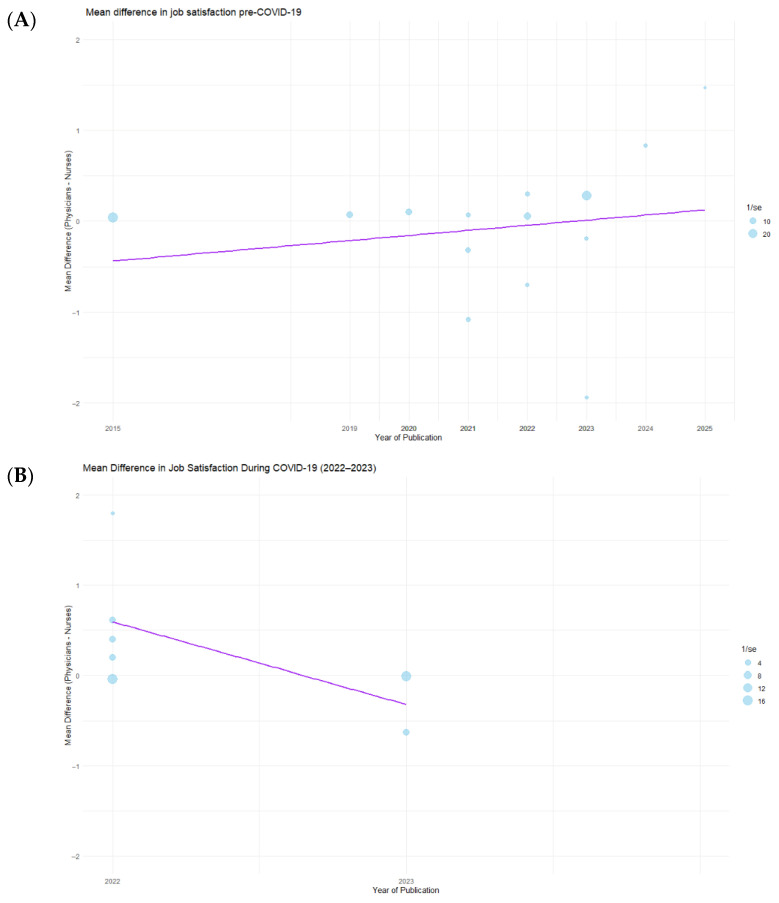

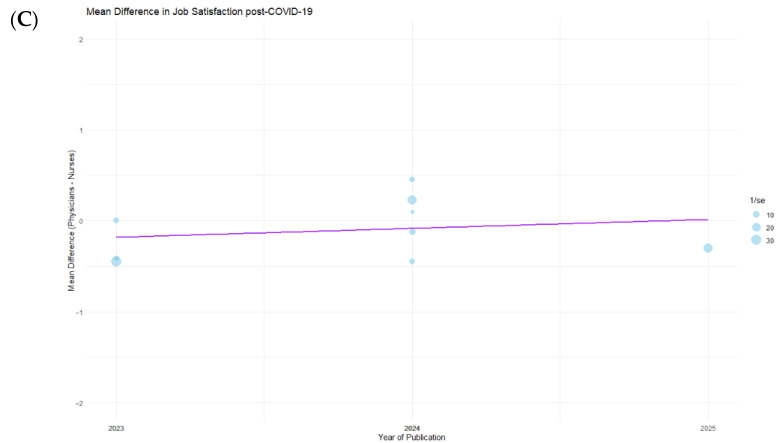
Meta-regression of job satisfaction differences between physicians and nurses across pandemic periods. Panel (**A**)—Pre-COVID-19 period; Panel (**B**)—COVID-19 period; Panel (**C**)—Post-COVID-19 period.

The results of the meta-regression analysis evaluating the association between the year of publication and the mean difference in job satisfaction between physicians and nurses are presented in Figure 3. Panel A shows data from the pre-COVID-19 period. The analysis revealed no statistically significant association between publication year and mean difference (*p* = 0.73), indicating that temporal trends did not explain the variability in job satisfaction differences before the pandemic. Panel B shows data from the COVID-19 period. The regression line demonstrated a downward trend in mean difference over time; however, this relationship was not statistically significant (*p* = 0.32). Panel C shows data from the post-COVID-19 period. Similarly, no statistically significant association was observed (*p* = 0.86), suggesting that publication year did not meaningfully influence job satisfaction differences after the pandemic.

### 3.4. Sensitivity Analysis

**Figure 4:**
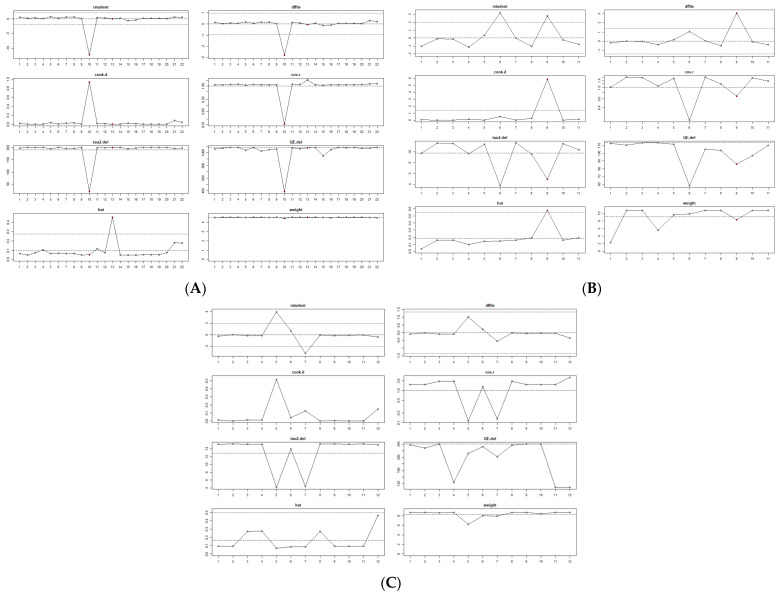
Influence analysis of individual studies across pandemic periods. Panel (**A**)—Pre-COVID-19 period; Panel (**B**)—COVID-19 period; Panel (**C**)—Post-COVID-19 period.

Sensitivity analyses, based on leave-one-out procedures, showed that no single study had a disproportionate impact on the pooled effect size, confirming the robustness and stability of the meta-analytic findings, as illustrated in Figure 4 (Panels A–C).

**Figure 5:**
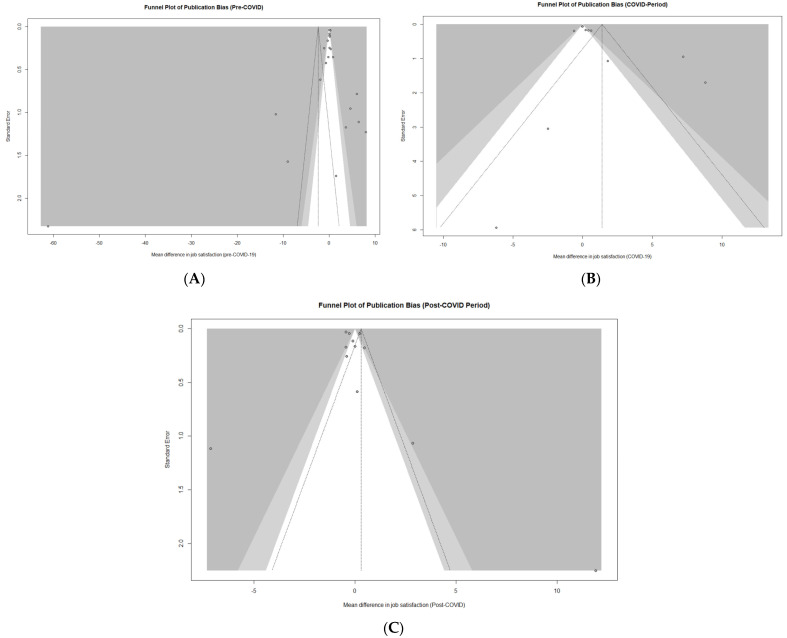
Funnel plots assessing publication bias across pandemic periods. Panel (**A**)—Pre-COVID-19 period; Panel (**B**)—COVID-19 period; Panel (**C**)—Post-COVID-19 period.

Publication bias assessment results are presented in Figure 5. Panel A shows the funnel plot for studies conducted before the COVID-19 pandemic. Visually the plot appeared asymmetrical, and, accordingly, Egger’s test indicated statistically significant asymmetry (z = −2.81, *p* = 0.0050). Panel B shows the funnel plot for studies conducted during the COVID-19 period. The plot appeared visually symmetrical, and Egger’s test confirmed the absence of significant asymmetry (z = −0.46, *p* = 0.65). Panel C presents the funnel plot for studies conducted after the COVID-19 pandemic. The plot appeared visually symmetrical, with most points evenly distributed around the pooled effect size. Egger’s test indicated borderline asymmetry (z = 1.89, *p* = 0.059), suggesting a possible small-study effect, although this did not reach conventional statistical significance (*p* < 0.05). Overall, these results imply a low likelihood of substantial publication bias in the post-COVID analysis. However, since fewer than 10 studies were available in each setting-stratified subgroup, the statistical power of Egger’s test was limited. Therefore, these results should be interpreted with caution.

### 3.5. Assessment of Bias Risk and Evidence Certainty

All studies incorporated into the current analysis achieved a minimum score of 6 out of 9 on the NOS, reflecting high methodological quality and a low likelihood of bias, as outlined in Table 3.

The results of the GRADE certainty assessment, as summarized in Table 4, indicate that the pooled mean differences in job satisfaction between physicians and nurses across all three time periods are characterized by low to very low certainty of evidence. These ratings were primarily influenced by high inconsistency across studies, imprecision of effect estimates, and potential publication bias. As such, the findings should be interpreted with caution.

## 4. Discussion

This systematic review and meta-analysis synthesized findings from diverse regions and clinical settings. Based on 38 cross-sectional studies of mostly moderate quality but very high heterogeneity, the certainty of evidence is low to very low. Overall, we found no consistent or clinically meaningful difference in job satisfaction between physicians and nurses across time periods. However, two setting-specific patterns emerged. During the pandemic, emergency care settings showed a small but statistically significant difference favoring physicians (SMD = 0.29), suggesting that contextual factors were particularly influential during peak pandemic stress. In the post-COVID period, mixed settings slightly favored physicians (SMD = 0.06), while primary care/outpatient settings favored nurses (SMD = −0.30). By national income strata, pre-COVID estimates indicated marginally higher satisfaction among physicians in high-income countries (SMD = 1.53) although subgroup tests across income levels were not statistically significant in any period.

Taken together, these results indicate that profession alone is an insufficient determinant of job satisfaction; rather, organizational context and historical period are salient modifiers. The small, setting-specific differences we observed likely reflect task structure, acuity, staffing ratios, and role autonomy that differ across emergency departments, hospitals, and ambulatory care. The transient advantage for physicians in emergency care during COVID-19 may be explained by more centralized decision authority, clearer role delineation during surge operations, or differential access to institutional resources [67,68,69]. Conversely, the post-COVID advantage for nurses in primary care could reflect redistribution of responsibilities, expansion of nursing roles in chronic disease management and vaccination, and stabilization of workflows as acute pandemic pressures receded [70].

More broadly, existing literature consistently links high workload and extended working hours to diminished job satisfaction, increased medical errors, and elevated burnout rates across healthcare professions [71,72]. Even where statistically significant, the observed effect sizes on job satisfaction differences were small, underscoring the predominance of shared determinants—such as collaboration, workload, staffing adequacy, leadership support, interprofessional climate, scheduling flexibility, and perceived safety—over profession-specific factors. Job satisfaction is fundamentally an affective and attitudinal construct, and across roles, it is strongly shaped by the quality of interprofessional collaboration [73]. Syntheses of nurse–physician collaboration emphasize the importance of shared decision-making, teamwork, and communication [74], and prior evidence links high-quality collaboration to improved patient outcomes, including reduced mortality [75]. Therefore, interventions aimed at strengthening these modifiable organizational levers are more likely to yield meaningful improvements in job satisfaction than those targeting individual professions in isolation.

The post-pandemic period represents a critical inflection point for healthcare workforce recovery. Effective planning should prioritize the integration of all clinicians into accountable, interprofessional primary care teams supported by robust infrastructure [76]. However, recovery must extend beyond structural reforms. Targeted investments in mental health support, flexible scheduling, and career development pathways are essential to address burnout and restore morale [77]. Health systems must also focus on rebuilding trust, fostering psychological safety, and ensuring transparent communication between leadership and frontline staff. Importantly, recovery strategies should be tailored to the specific needs of different care settings. Emergency departments may require surge-capacity planning and trauma-informed support, while primary care settings may benefit from expanded roles for nurses and community health workers to meet growing demands in chronic disease management and preventive care. Workforce resilience will depend not only on adequate staffing and resources but also on a renewed commitment to equity, recognition, and professional growth [78].

From a global health equity perspective, it is important to note that, among the 38 included studies, only two were conducted in lower-middle-income countries (LMICs) [55,56]. This limited representation restricts the generalizability of our findings to health systems operating under constrained resources. Workforce dynamics in LMICs are shaped by distinct challenges, including limited infrastructure, workforce migration, lower salary scales, and reduced access to psychosocial support [79,80]. These contextual factors may significantly influence job satisfaction but are underexplored in the current literature.

Practical implications of our study findings: (1) Health systems should develop profession- and setting-specific support programs. The needs of nurses in primary care likely differ from those of physicians in emergency departments. During system shocks (e.g., pandemics), emergency departments may require distinct support packages, whereas primary care may benefit from targeted physician-focused interventions in the recovery phase to close emerging gaps. (2) Leaders should prioritize improving the modifiable drivers of satisfaction within their specific clinical environments, such as workload management, autonomy, and interprofessional collaboration, rather than assuming inherent professional differences. (3) The changes observed across the three periods highlight the need for ongoing monitoring of workforce well-being as the healthcare landscape continues to evolve.

This review adhered to PRISMA guidance, used a registered protocol, applied random-effects models, conducted prespecified subgroup and meta-regression analyses, and assessed study quality with an adapted NOS tool. Nonetheless, several limitations warrant careful interpretation. First, all evidence was cross-sectional, precluding causal inference and susceptible to selection and reporting biases. Second, measurement non-equivalence across instruments required harmonization and likely contributed to heterogeneity. Third, the language restriction (English-only), strict outcome-based inclusion criteria without data imputation, and the post-2019 search window may have led to the exclusion of relevant studies. Fourth, setting classification relied on authors’ descriptions and may not fully capture within-setting variation in roles or workload. Fifth, while publication bias was explored, small-study effects cannot be ruled out. Sixth, our GRADE appraisal indicates low to very low certainty of evidence across the meta-analytic outcomes. Substantial heterogeneity was observed across nearly all pooled models (I^2^ ≈ 90–99%). This variability likely reflects differences in measurement instruments, health system structures, and the timing of data collection relative to the COVID-19 pandemic across countries. Although meta-regression by publication year and influence analyses were conducted, they did not fully account for the observed heterogeneity. This suggests the presence of unmeasured confounding factors, such as differences in participant demographics, compensation policies, nurse-to-patient ratios, electronic health record burdens, unionization status, and region-specific burnout dynamics. Additionally, there is a risk of bias—particularly selection bias—due to variation in recruitment strategies and sampling frames across studies. These differences may limit the representativeness and generalizability of the findings. Therefore, the results of our study should be interpreted with caution. Analyses stratified by country income level also revealed considerable heterogeneity, implying that intra-group variations in policy and organizational practices may outweigh differences between income groups. Lastly, the concept of job satisfaction remains loosely defined, with no universally accepted framework despite extensive scholarly inquiry [81]. Disagreement over its key determinants has led to varied methodological approaches across studies [81,82].

## 5. Conclusions

This systematic review and meta-analysis offer a comprehensive, longitudinal examination of job satisfaction differences between physicians and nurses across the critical phases of the COVID-19 pandemic. The findings reveal that job satisfaction is not solely determined by professional role but is significantly influenced by temporal and contextual factors. Prior to the pandemic, no consistent differences were observed. During the pandemic, emergency care settings showed a small but significant advantage for physicians, while post-pandemic trends revealed a modest divergence favoring nurses in primary care and physicians in mixed settings. Given the very high heterogeneity and low certainty, observed differences should be interpreted cautiously and not as definitive professional disparities.

The observed heterogeneity highlights the complexity of job satisfaction and suggests that profession and setting are only part of a broader constellation of influencing factors. Health systems should adopt nuanced, context-sensitive strategies to support workforce well-being. Future research should move beyond divided professional comparisons and employ multivariate, longitudinal designs to identify the organizational, psychological, and situational determinants that most effectively enhance job satisfaction. There is also a pressing need to expand such research into LMIC settings, which remain severely underrepresented in the literature. Such insights are essential for cultivating a resilient, engaged, and sustainable healthcare workforce. Due to the methodological limitations of this meta-analysis, including high heterogeneity, reliance on cross-sectional data, and very low/low certainty of evidence, these results should be interpreted with caution.

## Figures and Tables

**Table 1:** Eligibility criteria of study selection based on the PECOS framework.

PECOS Framework	Include	Exclude
Population	Physicians and nurses working in hospitals, emergency departments, outpatient clinics, community centers, primary care, or mixed healthcare settings	Studies conducted in dental clinics, or those involving only paramedics, residents, students, or other clinical staff
Exposure	Assessment of job satisfaction using either a single-item question or a validated job satisfaction questionnaire	Studies evaluating other types of satisfaction (e.g., compulsion satisfaction, job dissatisfaction, care satisfaction)
Comparator	Not applicable	Not applicable
Outcome	Primary outcome: Mean and standard deviation of job satisfaction scores reported separately for nurses and physicians. Secondary outcomes: (a) Care setting (hospital, emergency department, outpatient, primary care, mixed); (b) Time period (pre-COVID, during COVID, post-COVID)	Studies reporting only median and interquartile range scores, or the proportion of staff satisfied. Studies combining pre-COVID and COVID periods without separate analysis were also excluded.
Study design	Cross-sectional data from observational studies published in English	Randomized and non-randomized interventional studies, reviews, editorials, commentaries, and conference proceedings.

**Table 2:** Included study descriptions.

Last Name, Year	Country	Country Income Level	Study Design	Assessment Period	Assessment Setting	Group Descriptions	Physician Total	Nurse Total	Assessment Instrument
Pre-COVID									
Lin, 2015 [30]	China	upper-middle income	cross-sectional	December 2013 and April 2014	outpatients	-	569	564	Job satisfaction scale
Bebetsos, 2019 [31]	Greece	high income	cross-sectional	2018–2019	hospitals	-	112	310	The Safety Attitudes Questionnaire
Koch, 2020 [32]	Germany	high income	cross-sectional	September 2017	hospital	-	855	205	Copenhagen Psychosocial Questionnaire
Peter, 2020 [33]	Switzerland	high income	cross-sectional	September 2017 until the end of March 2018	acute care and rehabilitation hospitals	-	299	1905	Copenhagen psychosocial questionnaire
Siebenhüner, 2020 [34]	Switzerland	high income	cross-sectional	2015 and 2016	hospital	-	222	705	Single-item question
Wagner, 2020 [35]	Germany	high income	cross-sectional	2015	hospital	G-COPSOQ III (a) G-COPSOQ II (b)	381 (a) 381 (b)	567 (a) 567 (b)	Copenhagen psychosocial questionnaire G-COPSOQ III G-COPSOQ II
Connell, 2021 [36]	Australia	high income	cross-sectional	December 2017	emergency department	-	25	100	Safety Climate Survey
House, 2021 [37]	USA	high income	cross-sectional	21 January 2020 to 11 March 2020	hospitals in military health system	-	36	217	Single-item question
Negri, 2021 [38]	Italy	high income	cross-sectional	January 2020	outpatient	-	53	55	Job Satisfaction Questionnaire
Zikusooka, 2021 [39]	Türkiye	upper-middle income	cross-sectional	October and November 2019	outpatient	general physician (a); specialist physician (b)	259 (a) 77 (b)	555 (a) 555 (b)	Job Satisfaction Survey
Leong, 2022 [40]	Malaysia	upper-middle income	cross-sectional	June and August 2019	hospital	-	160	304	Safety Attitudes Questionnaire
Mundt, 2022 [41]	USA	high income	cross-sectional	1 July 2013 to 31 December 2014	primary care	male physicians (a); female physicians (b)	10 (a) 14 (b)	27 (a) 27 (b)	Warr–Cook–Wall job satisfaction survey
Poghosyan, 2022 [42]	USA	high income	cross-sectional	Summer and fall of 2017	primary care	-	186	398	Single-item question
Cunningham, 2023 [43]	USA	high income	cross-sectional	2015	hospital, outpatient and community centers	-	3941	2221	Single-item question
Hasebrook, 2023 [44]	Germany	high income	cross-sectional	2017	emergency care	-	57	23	Job Satisfaction Survey
Ning, 2023 [45]	China	upper-middle income	cross-sectional	January 2020	primary care	-	277	243	Job Description Index Scale
Zhang, 2024 [46]	China	upper-middle income	cross-sectional	November 2019 and January 2020	hospitals	-	303	217	Brayfield and Rothe’s job satisfaction questionnaire
Liu, 2025 [47]	China	upper-middle income	cross-sectional	August 2019	primary care, general medical practitioners (a); public health physicians (b)	-	193 (a) 86 (b)	492 (a) 492 (b)	Minnesota Job Satisfaction Scale
COVID									
Alfuraih, 2022 [48]	Saudi Arabia	high income	cross-sectional	June and July 2020	Hospital—radiographers, ICU nurses (a); department nurses (b)	-	412 (a) 412 (b)	190 (a) 308 (b)	Job Satisfaction Survey
Diakos, 2022 [49]	Greece	high income	cross-sectional survey	August to October 2021	emergency department	-	34	39	Job Satisfaction Survey
Goula, 2022 [50]	Greece	high income	cross-sectional	15 May 2021 to 15 September 2021	hospital	-	182	74	Job Satisfaction Tool
Huang, 2022 [51]	China	upper-middle income	cross-sectional survey	1 July to 30 September 2020	hospital and community health center	-	373	530	Job Satisfaction Scale
Jonker, 2022 [52]	South Africa	upper-middle income	cross-sectional	24 November 2020 and 24 March 2021	emergency department	physicians CHBAH (a); physicians CMJAH (b)	19 (a) 19 (b)	21 (a) 19 (b)	Job Satisfaction Survey
Akgun, 2023 [53]	Türkiye	upper-middle income	cross-sectional	December 2021 and January 2022	hospital—gynecology department	-	29	22	Minnesota Job Satisfaction Scale
Batarfi, 2023 [54]	Saudi Arabia	high income	cross-sectional	11 November 2021 until 11 March 2022	emergency department	-	29	50	20-question questionnaire on job satisfaction
Mouaddib, 2023 [55]	Morocco	lower-middle income	cross-sectional	18 January 2021 to 31 March 2021	primary care	-	138	187	Warr–Cook–Wall job satisfaction scale
Chen, 2024 [29]	Taiwan	high income	cross-sectional	2021	hospital	COVID (a); post-COVID (b)	163	903	Taiwan Patient Safety Culture Survey
Post-COVID									
Deshmukh, 2023 [56]	India	lower-middle income	cross-sectional	2022	hospital	-	160	120	Job Satisfaction Survey Scale
Plantinga, 2023 [57]	USA	high income	cross-sectional	26 September 2022, and 22 October 2022	hospital and outpatient dialysis centers	-	89	174	Single-item question
Shi, 2023 [58]	China	upper-middle income	cross-sectional	27 July to 27 September 2021	hospitals	-	920	986	Minnesota Satisfaction Questionnaire and the Ask-Form Employee Satisfaction questionnaire
Cascales-Martinez, 2024 [59]	Spain	high income	cross-sectional	1 October and 1 December 2021	emergency care	-	57	28	Minnesota Satisfaction Questionnaire—general satisfaction
Chen, 2024 [29]	Taiwan	high income	cross-sectional	2022	hospital	COVID (a); post-COVID (b)	87	787	Taiwan Patient Safety Culture Survey
Fernández-Martínez, 2024 [60]	Spain	high income	cross-sectional	March and April 2023	hospital, primary care and community centers	-	82	52	UNIPSICO test battery
Jolidon, 2024 [61]	Switzerland	high income	cross-sectional	2022	hospital, clinic and community	-	212	533	Single-item question
Maniscalco, 2024 [62]	Belgium, Netherlands, Italy and Poland	high income	cross-sectional	16 May and 30 September 2022	hospital	-	381	1351	Copenhagen psychosocial questionnaire
Puticiu, 2024 [63]	Romania	high income	cross-sectional	December 2023 and February 2024	emergency care	-	41	74	Single-item question
Sheekha, 2024 [64]	Canada	high income	cross-sectional	December 2021 and April 2022	hospital, clinic and community	-	380	1053	Job Satisfaction Survey
Wang, 2024 [65]	China	upper-middle income	cross-sectional	1 August and 31 December 2022	anesthesia medical staff’	-	989	122	Minnesota Satisfaction Questionnaire
Williams, 2025 [66]	England	high income	cross-sectional	June 2022	general practice	-	1933	2055	Single-item question

Abbreviations: CHBAH—Chris Hani Baragwanath Academic Hospital; CMJAH—Charlotte Maxeke Johannesburg Academic Hospital; G-COPSOQ II—German version of Copenhagen Psychosocial Questionnaire second version; G-COPSOQ III—German version of Copenhagen Psychosocial Questionnaire third version; ICU—intensive care unit.

**Table 3:** Assessment of Risk of Bias.

Study	Selection	Comparability	Exposure	Total
Pre-COVID				
Lin, 2015 [30]	5	1	2	7
Bebetsos, 2019 [31]	4	1	2	7
Koch, 2020 [32]	5	1	2	8
Peter, 2020 [33]	4	1	2	7
Siebenhüner, 2020 [34]	4	1	2	7
Wagner, 2020 [35]	4	1	2	7
Connell, 2021 [36]	4	1	2	7
House, 2021 [37]	4	1	2	7
Negri, 2021 [38]	4	1	2	7
Zikusooka, 2021 [39]	4	1	2	7
Leong, 2022 [40]	4	1	2	7
Mundt, 2022 [41]	4	1	2	7
Poghosyan, 2022 [42]	4	1	3	8
Cunningham, 2023 [43]	5	1	2	8
Hasebrook, 2023 [44]	4	1	2	7
Ning, 2023 [45]	4	1	2	7
Zhang, 2024 [46]	5	1	2	8
Liu, 2025 [47]	4	1	2	7
COVID				
Alfuraih, 2022 [48]	4	1	3	8
Diakos, 2022 [49]	5	1	2	8
Goula, 2022 [50]	4	1	2	7
Huang, 2022 [51]	4	1	3	8
Jonker, 2022 [52]	5	1	2	8
Akgun, 2023 [53]	4	1	2	7
Batarfi, 2023 [54]	4	1	3	8
Mouaddib, 2023 [55]	5	1	2	8
Chen, 2024 [29]	4	1	2	7
Post-COVID				
Deshmukh, 2023 [56]	4	1	2	7
Plantinga, 2023 [57]	5	1	2	8
Shi, 2023 [58]	4	1	2	7
Cascales-Martinez, 2024 [59]	4	1	3	8
Chen, 2024 [29]	5	1	2	8
Fernández-Martínez, 2024 [60]	4	1	2	7
Jolidon, 2024 [61]	4	1	2	7
Maniscalco, 2024 [62]	5	1	2	8
Puticiu, 2024 [63]	4	1	2	7
Sheekha, 2024 [64]	4	1	2	7
Wang, 2024 [65]	5	1	2	8
Williams, 2025 [66]	4	1	3	8

**Table 4:** Evaluation of the certainty of evidence using GRADE framework.

Outcome	Risk of Bias	Inconsistency	Indirectness	Imprecision	Publication Bias	Certainty of Evidence
	Downgrading Score					
Mean difference pre-COVID	0	−2	0	−1	−1	Very low
Mean difference COVID	0	−2	0	−1	0	Low
Mean difference post-COVID	0	−2	0	−1	−1	Very low

## Data Availability

The original contributions presented in this study are included in this article. Further inquiries can be directed to the corresponding author.

## Supplementary Materials

The following supporting information can be downloaded at: https://www.mdpi.com/article/10.3390/healthcare13233008/s1, Figure S1. Meta-analysis of job satisfaction scores by country income level. Table S1. The complete PubMed search string.

## Abbreviations

The following abbreviations are used in this manuscript:

CHBAH	Chris Hani Baragwanath Academic Hospital
CI	Confidence interval
CINAHL	Cumulative Index to Nursing and Allied Health Literature
CMJAH	Charlotte Maxeke Johannesburg Academic Hospital
COPSOQ	Copenhagen Psychosocial Questionnaire
COVID-19	Coronavirus disease 2019
G-COPSOQ II	German version of COPSOQ II
G-COPSOQ III	German version of COPSOQ III
GRADE	Grading of Recommendations Assessment, Development and Evaluation
ICU	Intensive care unit
I^2^	I-squared heterogeneity statistic (inconsistency)
JSS	Job Satisfaction Survey
MSQ	Minnesota Satisfaction Questionnaire
NOS	Newcastle–Ottawa Scale (adapted for cross-sectional studies)
PECOS	Population, Exposure, Comparator, Outcome, Study design
PRISMA	Preferred Reporting Items for Systematic Reviews and Meta-Analyses
PROSPERO	International Prospective Register of Systematic Reviews
R	R programming language
RStudio	Integrated development environment (IDE) for R
SAQ	Safety Attitudes Questionnaire
SD	Standard deviation
SMD	Standardized mean difference
τ^2^	Between-study variance (tau-squared)
UNIPSICO	Psychosocial Risks in Organizations test battery (UNIPSICO)
