# Supplementary material for: Temporal and Contextual Variations in Job Satisfaction Between Physicians and Nurses: A Systematic Review and Meta-Analysis
Source: Healthcare (Basel). 2025 Nov 21;13(23):3008. doi: 10.3390/healthcare13233008 (PMC12692353; doi:10.3390/healthcare13233008)
Supplement: Supplementary file 1 [file healthcare-13-03008-s001.zip › Supplementary Figure S1.pdf]

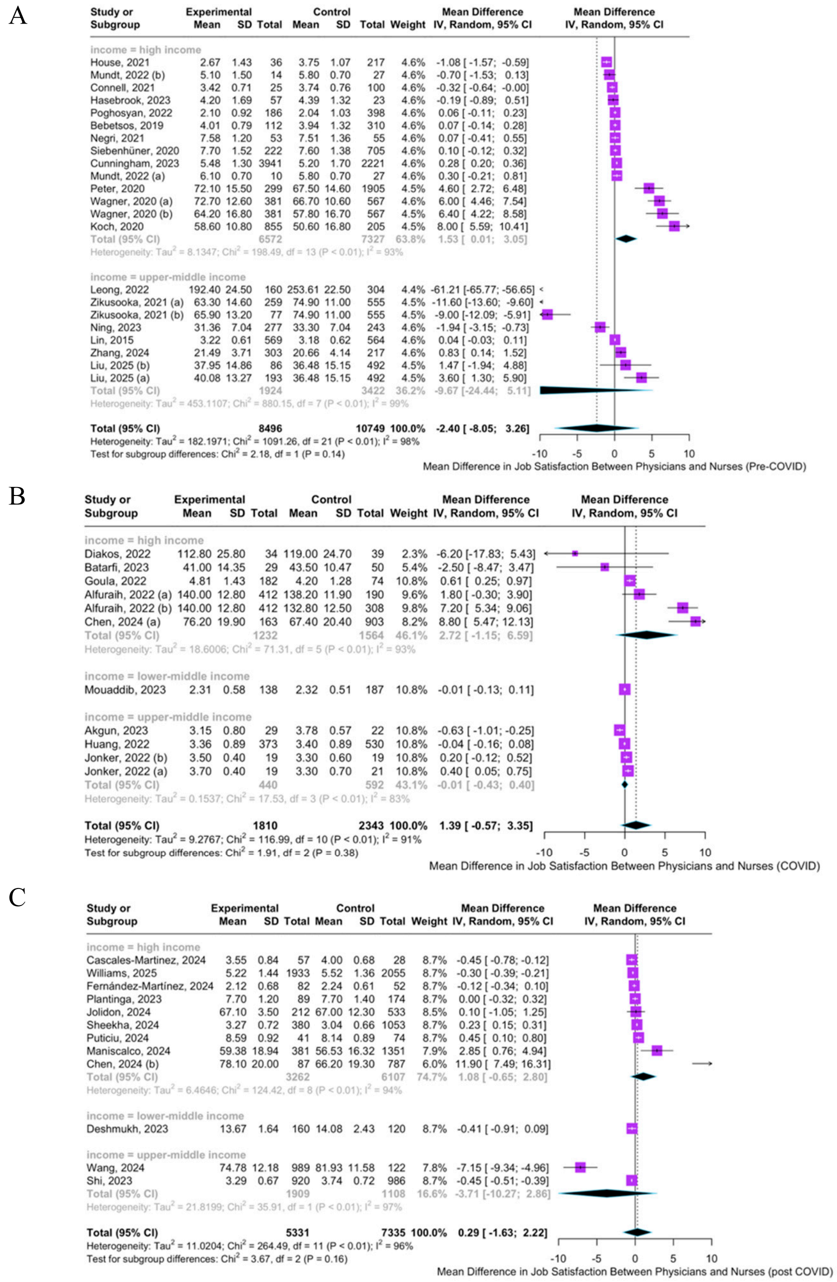

Figure S1. Forest plots comparing mean differences in job satisfaction between physicians and nurses across pandemic periods by country income level: A) Pre-COVID-19 period; B) COVID-19 period; C) Post-COVID-19 period.
